# Off-plane technique ultrasound-guided pericardiocentesis via anterior approach: short communication

**DOI:** 10.1186/s13089-024-00383-4

**Published:** 2024-06-24

**Authors:** David Rene Rodriguez Lima, Laura Otálora González, Felipe Noriega Acosta

**Affiliations:** 1 Critical and Intensive Care Medicine, Hospital Universitario Mayor-Méderi, Bogotá, Colombia; 2 Grupo de Investigación Clínica, Escuela de Medicina y Ciencias de la Salud, Universidad del Rosario, Bogotá, Colombia; 3 Escuela de Medicina y Ciencias de la Salud, Universidad del Rosario, Bogotá, Colombia; 4 Cirugía Cardiovascular, Hospital Universitario Mayor-Méderi, Bogotá, Colombia

**Keywords:** Pericardiocentesis, Critical care, Bedside ultrasound

## Abstract

The pericardiocentesis procedure is common, often performed via the subxiphoid approach, although other transthoracic approaches have been described. This short communication describes an off-plane technique ultrasound-guided pericardiocentesis using an anterior approach, performed using a linear transducer and guided in real-time by ultrasound, offering the advantage of continuous needle tracking to reduce complications associated with this approach such as pneumothorax, inadvertent cardiac puncture, and injury to the left internal mammary artery (LIMA).

## Introduction

The pericardium is a relatively rigid sac that envelops the heart and the roots of the great vessels, working as a point of fixation and physical barrier for the heart [1]. It consists of two layers: a parietal layer, which is fibrous, and a visceral layer, which is a serous membrane. The space between these two layers is known as the intrapericardial space, which contains about 20–25 ml under normal conditions [2]. Pericardial effusion is defined as the abnormal accumulation of fluid in the pericardial cavity, which can develop due to overproduction of pericardial fluid, decreased reabsorption, or an imbalance between the hydrostatic and oncotic pressures of the fluid [3]. There are multiple causes for the development of pericardial effusion such as heart failure, trauma, neoplasms, infections, connective tissue diseases, and metabolic disorders [4].

Pericardial effusion, depending on its volume and time of onset, can lead to increased intrapericardial pressures transmitted to the cardiac cavities, leading to hemodynamic alterations and in severe cases, being associated with shock, a condition known as cardiac tamponade [5, 6]. The diagnosis of pericardial effusion is clinical, supported by echocardiographic findings suggesting alterations in intracardiac filling pressures. In cases of hemodynamic repercussion, shock, or unknown etiology of the effusion, drainage should be performed. The most commonly employed technique is percutaneous pericardiocentesis [7, 8]. Traditionally, the approach to performing this procedure has been based on anatomy or guided by fluoroscopy. However, with the advancement of technology, ultrasound has provided the opportunity to visualize pericardial effusion and adjacent structures, which has led to the development of different techniques and approaches for performing this procedure. Therefore, ultrasound-guided pericardiocentesis should be considered the “gold standard” in contemporary practice [9].

The subxiphoid approach is the most employed technique; however, during this procedure, it is difficult to guide the puncture in real time due to the anatomy and position of the needle. Ultrasound ends up being more of a tool for confirming intrapericardial position rather than real-time guidance [2]. Approaches for pericardiocentesis via anterior, subcostal in-plane [6], and anterior in-plane routes have been described [10]. In this study, we aim to describe an anterior off-plane technique that allows for continuous evaluation of the pleura and the anatomical location of the left internal mammary artery (LIMA) to avoid pneumothorax and inadvertent puncture of the LIMA.

## Technique

This technique is demonstrated in a 63-year-old patient with a history of breast cancer who presents with symptoms of dyspnea and cough, and echocardiography reveals signs of cardiac tamponade secondary to malignant pericardial effusion.

Pericardiocentesis via anterior off-plane approach guided by real-time echocardiography is described as follows, a SonoSite M-Turbo ultra- sound machine with a 5-MHz small-footprint convex transducer and a 13-MHz linear transducer were used.

**Fig. 1:**
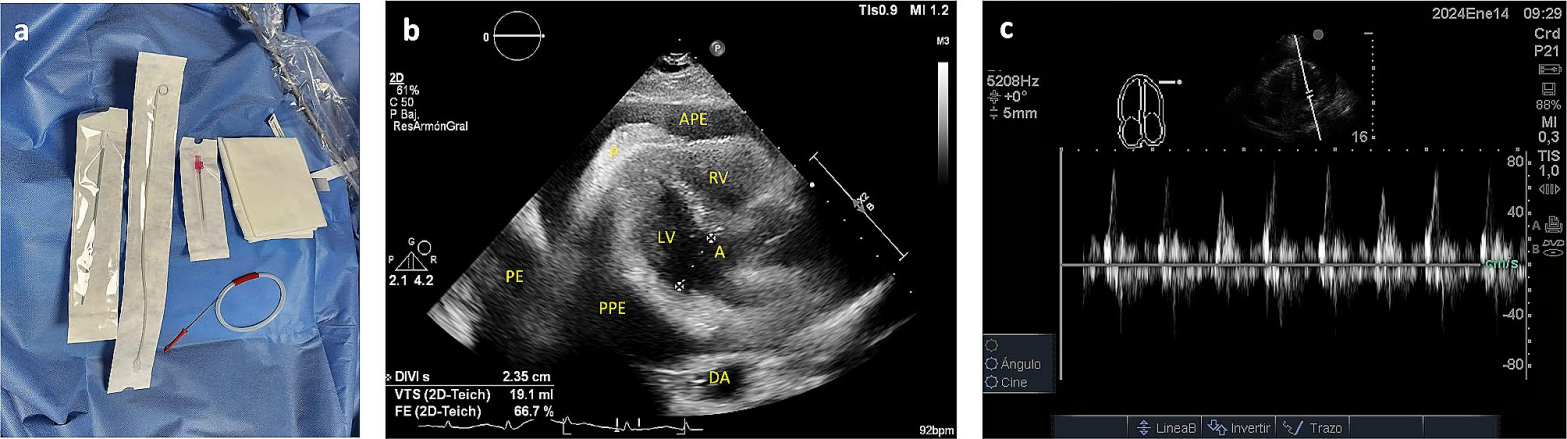
(**a**) Pericardiocentesis kit (**b**) Long axis window showing anterior and posterior pericardial effusion. APE: Anterior pericardial effusion, RV: right ventricle, LV: left ventricle, A: aorta, PE: pleural effusion, PPE: posterior pericardial effusion, DA: descending aorta, P: pericardium thickness (**c**) Doppler through mitral valve showing changes in peak E-wave (> 25%) during respiration

**Fig. 2:**
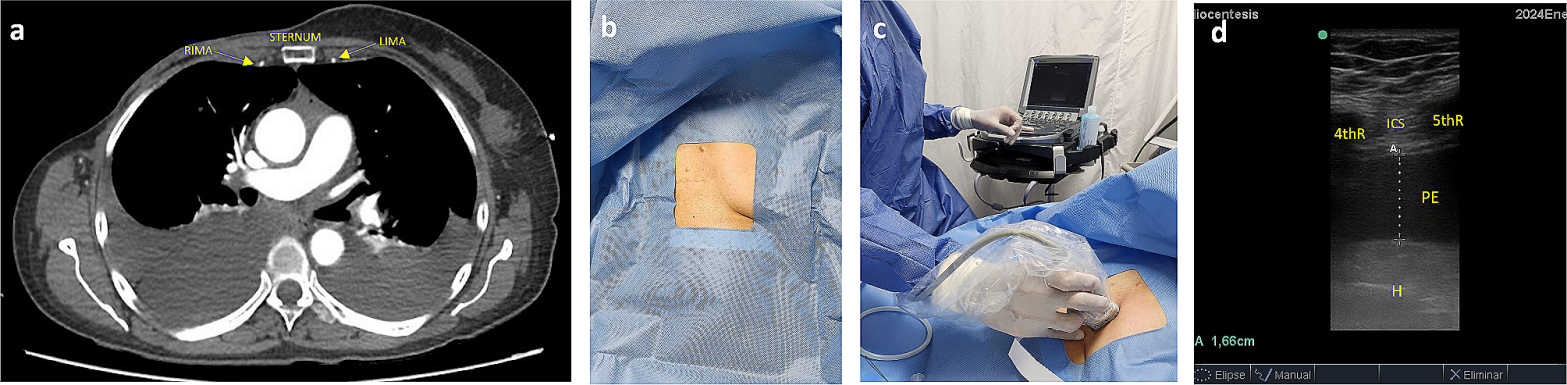
(**a**) CT scan showing LIMA and RIMA being laterally around 1.5 mm from the sternum. CT: computed tomography, RIMA: right internal mammary artery, LIMA: left internal mammary artery (**b**) Asepsis and antisepsis before the procedure (**c**) Linear transducer in longitudinal plane (**d**) High frequency linear transducer showing the anterior pericardial effusion. ICS: inter costal space, PE: pericardial effusion, H: heart, 4thR: 4th rib, 5thR: 5th rib

**Fig. 3:**
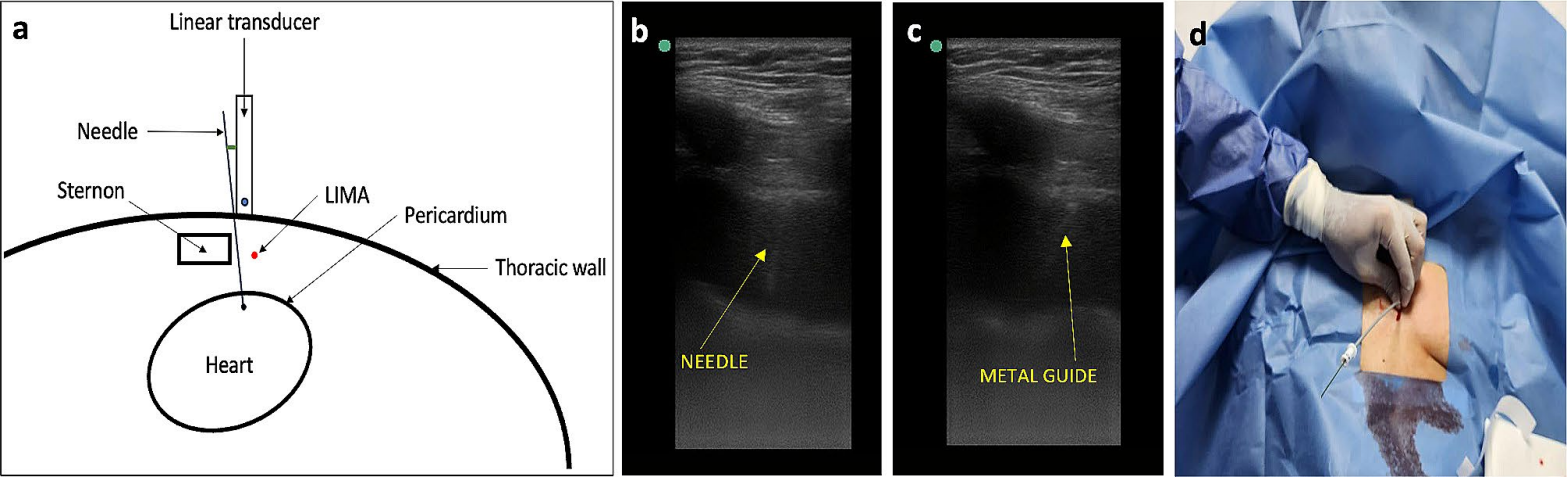
(**a**) Diagram of the puncture technique, it must be performed at the center of the transducer with minimal inclination (green angle) to allow the visualization of the needle throughout the trajectory. LIMA: Left internal mammary artery. (**b**) The puncture is made off-plane and guided in real-time until its entry into the pericardium (**c**) The metal guide is passed to the pericardium and verified in real time (**d**) 8.5Fr dilatator is inserted 1–3 cm

**Fig. 4:**
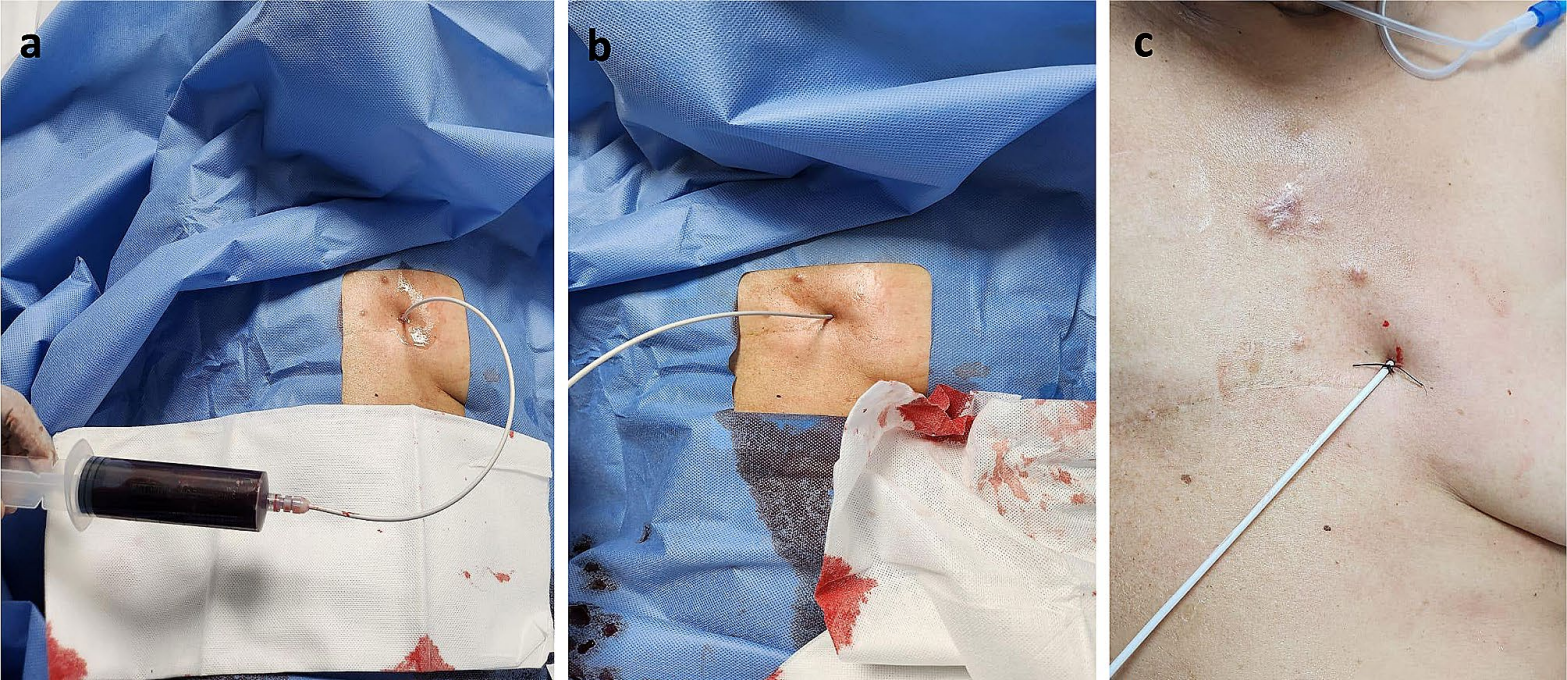
(**a**) Pigtail catheter is inserted up to10cm, ensuring no hole remains outside the thorax, return is verified with syringe aspiration (**b**) Catheter connected to a drainage bag (**c**) Secure catheter with 2 − 0 silk

**Fig. 5:**
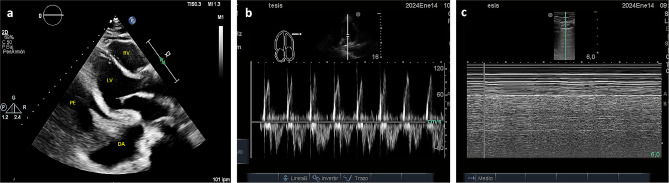
(**a**) Echocardiographic evaluation after drainage with a decrease in the effusion RV: right ventricle, LV: left ventricle, PE: pleural effusion, DA: descending aorta (**b**) Doppler through mitral valve showing no changes in peak E-wave during respiration. (**c.**) Absence of pneumothorax being evaluated by assessing pleural sliding and the presence of the seashore sign in the nearest pleural space that can be visualized

**Fig. 6:**
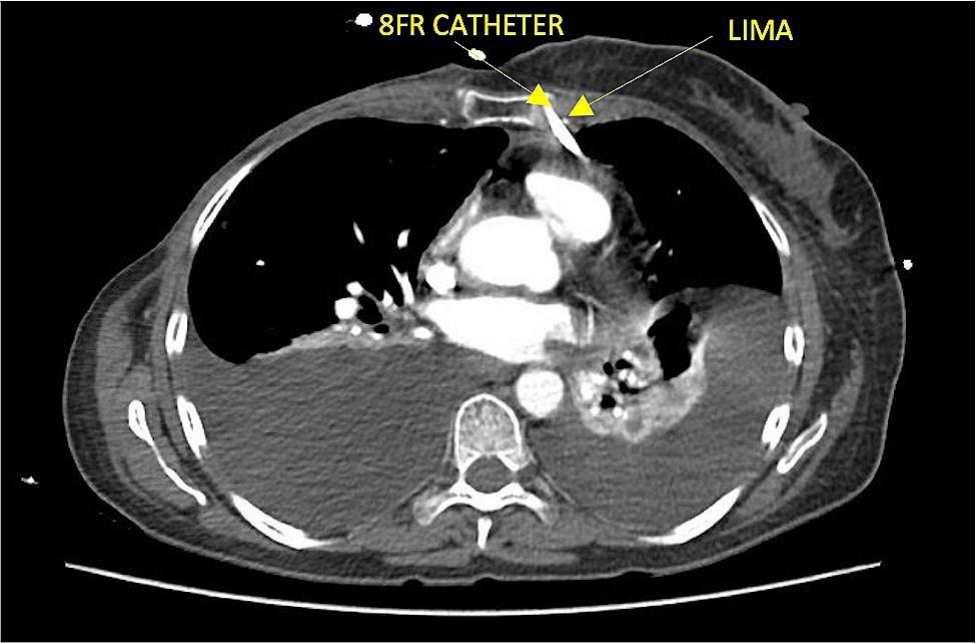
CT scan showing distance between the catheter passage and the LIMA. CT: computed tomography, LIMA: left internal mammary artery

1. The pericardiocentesis kit is prepared, it contains a 16-gauge thoracentesis needle, an 8.5 Fr dilator, an 8.0 Fr catheter, and a metal guide (Fig. 1a).
2. The patient is placed in the supine position with the head elevated between 30 and 40 degrees.
3. Echocardiographic assessment is performed at the patient’s bedside using the four basic windows: long axis, short axis, apical four-chamber, and subxiphoid views. The most important window in this procedure is the long-axis view, where the pericardial effusion should be anterior (between the thoracic wall and the right cavities) (Fig. 1b), if there is not anterior pericardial effusion, this technique is contraindicated; also it is recommended to assess hemodynamic repercussion by evaluating transmitral variability (Fig. 1c).
4. If fluid is observed in the anterior space with a low frequency convex transducer, its distance between the thoracic wall and the pericardium should be measured with a high frequency linear transducer, to consider anterior approach it should be greater than 15 mm in diastole. The 4th and 6th intercostal spaces should also be evaluated, and the widest space should be selected (Fig. 2c and d).
5. It must be ensured that the LIMA is not in the puncture trajectory. The LIMA has been described to be laterally around 1.47 +/- 0.30 cm from the sternum [11] (Fig. 2a) and it is recommended whenever possible to visualize the LIMA prior to needle entry for a safer puncture [12, 13]; in our experience, Doppler assessment is not always able to clearly show its location. Therefore, for this technique, it is suggested that the transducer be placed longitudinally parallel to the sternum as close as possible and not beyond 1 cm of it (Fig. 2c). Doppler color should also be used to rule out the presence of any vessel in the puncture trajectory.
6. After asepsis and antisepsis, with the linear transducer in longitudinal plane (Fig. 2b, c and d), the puncture is made off-plane and guided in real-time until its entry into the pericardium (Fig. 3a and b). The puncture is performed at the center of the transducer with minimal inclination to allow the visualization of the needle throughout the trajectory until reaching the pericardium (Fig. 3a). It has been described that a greater angle of needle entry results in less pronounced scattering artifact; however, the needle tip may appear less visible when entering the pericardium, potentially causing inadvertent puncture of the heart due to this effect [14]. To prevent inadvertent punctures, we recommend maintaining negative pressure with the syringe once the skin is pierced and stopping the advancement of the needle once fluid return is obtained. Additionally, it is important to measure the thickness of the thoracic wall to have a preliminary idea of the distance the needle needs to be inserted prior to the puncture.
7. Once the needle is positioned (Fig. 3b), the metal guide is passed, and its entry into the pericardium is verified (Fig. 3c). It is recommended to insert the guide 5–7 cm, depending on the thoracic thickness.
8. Once it is confirmed that the guide is in the pericardium, an 8.5 Fr dilator is inserted 1–3 cm, depending on the thoracic thickness (Fig. 3d).
9. The dilator is removed, and an 8 Fr pigtail catheter is inserted up to 10 cm, ensuring that no hole remains outside the thorax. Return is verified, and it is connected to a drainage bag or a Pleur-Evac drainage system (Fig. 4a and b).
10. If samples are required, it is recommended to connect to a three-way stopcock to have a route for sample collection, while the drainage is left in place.
11. It is recommended to secure with 2 − 0 silk (Fig. 4c).
12. Once at least 200 cc have been drained, a new echocardiographic evaluation is recommended, observing a decrease in effusion (Fig. 5a) and evaluating transmitral flow to assess changes in hemodynamic repercussion (Fig. 5b).
13. Finally, the absence of pneumothorax should also be evaluated by assessing pleural sliding and the presence of the seashore sign in the nearest pleural space that can be visualized (Fig. 5c).
14. In this patient, pericardial thickening suggestive of neoplastic etiology of the effusion is observed, so a chest tomography is performed to show how close the distance is between the catheter passage and the LIMA (Fig. 6).

This technique is similar to that described by Osman et al. [7] for sternal in-plane pericardiocentesis. Osman recommends a needle entry inclination of 45 degrees for visualization throughout its trajectory; however, if the thickness of the thoracic wall is 1.5–2.5 cm, as in the case of this patient, theoretically, the needle must cross this same distance in the horizontal plane with the potential risk of puncture of the LIMA, especially when adequate visualization of it by Doppler is not possible.

Our research group previously presented a series of ultrasound-guided thoracentesis performed off-plane, where the complication rate was 1.2%, with no vascular injury complications [15]. It is clarified that the use of off-plane techniques requires excellent hand control and probe-needle coordination, especially with very long pericardiocentesis needles. Therefore, it is recommended that this procedure be performed by personnel experienced in ultrasound-guided procedures.

## Conclusion

The off-plane anterior pericardiocentesis approach offers the advantage of never losing sight of the needle tip, unlike other approaches, especially the subxiphoid approach, where observing needle entry into the pericardium in obese patients is highly challenging. This enables us to minimize the risk of pneumothorax, inadvertent cardiac puncture, and injury to the LIMA.

## Data Availability

All data used during this study are available by email at the request of the editorial committee.

## Abbreviations

4thR: 4th rib
5thR: 5th rib
A: Aorta
APE: Anterior pericardial effusion
CT: Computed tomography
DA: Descending aorta
H: Heart
ICS: Inter costal space
LIMA: Left internal mammary artery
LV: Left ventricle
P: Pericardium thickness
PE: Pleural effusion
PPE: Posterior pericardial effusion
RIMA: Right internal mammary artery
RV: Right ventricle
